# Supplementary material for: Preclinical Characterization of the 177Lu-Labeled Prostate Stem Cell Antigen (PSCA)-Specific Monoclonal Antibody 7F5
Source: Int J Mol Sci. 2023 May 29;24(11):9420. doi: 10.3390/ijms24119420 (PMC10253363; doi:10.3390/ijms24119420)
Supplement: Supplementary file 1 [file ijms-24-09420-s001.zip › ijms-2394048-supplementary.pdf]

Supplementary Materials

Preclinical characterization of the ^{177}Lu-labeled prostate stem cell antigen (PSCA)-specific monoclonal antibody 7F5

Franziska Striese [1,2†], Sandy Gräßel [1,2†], Christin Neuber [1†], Claudia Arndt [1†], Martin Ullrich [1], Jörg Steinbach [1,2], Jens Pietzsch [1,2], Ralf Bergmann [1,3], Hans-Jürgen Pietzsch [1], Wiebke Sihver [1*], Marcus Frenz [4], Anja Feldmann [1], and Michael P. Bachmann [1,5,6*]

[1] Helmholtz-Zentrum Dresden-Rossendorf, Institute of Radiopharmaceutical Cancer Research, 01328 Dresden, Germany; f.striese@hzdr.de (F.S.); s.weissflog@hzdr.de (S.W.); w.sihver@hzdr.de (W.S.); a.feldmann@hzdr.de (A.F.); c.arndt@hzdr.de (C.A.); m.ullrich@hzdr.de (M.U.); j.steinbach@hzdr.de (J.S.); j.pietzsch@hzdr.de (J.P.); r.bergmann@hzdr.de (R.B.); h.j.pietzsch@hzdr.de (H.-J.P.); m.bachmann@hzdr.de (M.P.B.)

[2] Technical University Dresden, School of Science, Faculty of Chemistry and Food Chemistry, 01062 Dresden, Germany

[3] Semmelweis University, Institute of Biophysics and Radiation Biology, 1094 Budapest, Hungary

[4] Faculty of Informatik and Wirtschaftsinformatik, Provadis School of International Management and Technology AG, 65926 Frankfurt, Germany.

[6] National Center for Tumor Diseases (UCC/NCT), partner site Dresden, 01307 Dresden, Germany

[7] German Cancer Consortium (DKTK), partner site Dresden and German Cancer Research Center (DKFZ), 69120 Heidelberg, Germany

[†] These authors have contributed equally to this work and share first authorship

* Correspondence: m.bachmann@hzdr.de; Tel.: +49-351-260-3170 (M.P.B.); w.sihver@hzdr.de; Tel.: +49-351-260-2424 (W.S.)

Table of content

Figure S1. (A), (B), (C) Depiction of the IRF of [^{177}Lu]Lu-DTPA-7F5 on PSCA-negative PC3 cells.

Figure S2. (A) Time-dependent uptake of [^{177}Lu]Lu-DTPA-7F5 on PSCA-negative PC3 cells. (B) Saturation binding with [^{177}Lu]Lu- DTPA-7F5 on PSCA-negative PC3 cells.

Table S1. Biodistribution of [^{177}Lu]Lu-DTPA-7F5 in PC3-P tumor-bearing mice 1 and 48 h after injection, accumulation calculated as standard uptake value (SUV).

Table S2. Biodistribution of [^{177}Lu]Lu-DTPA-7F5 in PC3-P tumor-bearing mice, 1 and 48 h after injection, accumulation determined as ID/g tissue.

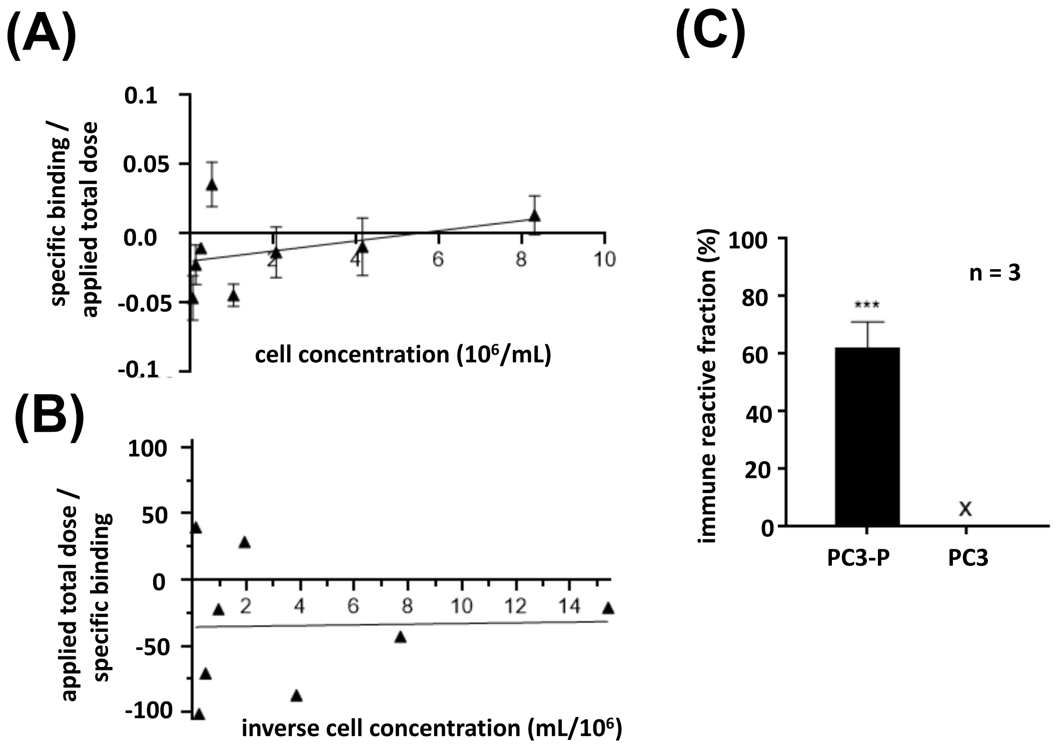

Figure S1. Estimation of the immunoreactive fraction (IRF) of [^{177}Lu]Lu-DTPA-7F5 on PSCA-negative PC3 cells. The radiolabeled Ab was incubated with increasing amount of PC3 cells at 37°C for 2 h. Thereafter, the cells were isolated by filtration through a glass fiber filter, that was counted in a gamma counter. (A) The ratio of specific binding to applied total activity was plotted against the cell concentration. (B) Totally applied activity divided through the specifically bound activity on the cells was plotted against the reciprocal cell concentration. The Y intercept gives the reciprocal of the IRF, not determinable in this case, since there was no binding of the radiolabeled 7F5. (C) The bar graph illustrates the IFR determined on PC3-P and PC3 cells.

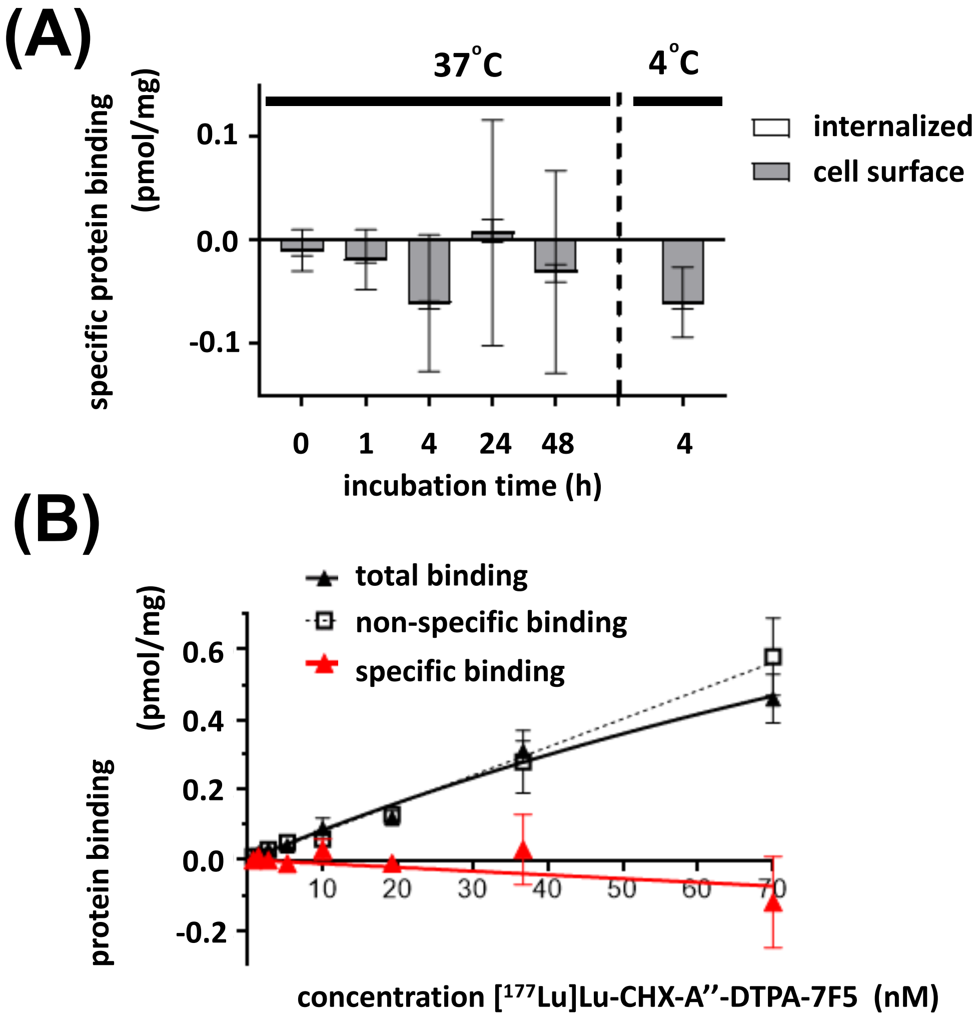

Figure S2. (A) Time-dependent uptake of [^{177}Lu]Lu-DTPA-7F5 on viable PSCA-negative PC3 cells. The samples were incubated at 37°C; at indicated time points the specific part as a percentage of the total radioactivity applied and the incorporated radioactivity were determined, respectively. (B) Saturation binding with [^{177}Lu]Lu-DTPA-7F5 on intact PSCA-negative PC3 cells. The cells were incubated for 2 h at 37°C with increasing [^{177}Lu]Lu-DTPA-7F5 concentrations. Nonspecific binding was determined in the presence of 0.5 µM of non-labeled anti-PSCA mAb 7F5. Red is the fitted line after subtracting the values of the nonspecific binding from those of the total binding (n = 3).

Table S1. Biodistribution of [^{177}Lu]Lu-DTPA-7F5 in PC3-P tumor-bearing mice 1 and 48 h after injection, accumulation calculated as standard uptake value (SUV).

	1 h	48 h
Blood	6.6 ± 0.3	1.6 ± 0.1***
Heart	2.6 ± 0.4	0.5 ± 0.1**
Brain	0.2 ± 0.0	0.1 ± 0.0***
Lungs	3.2 ± 0.6	0.8 ± 0.1**
Liver	3.1 ± 0.4	4.7 ± 0.4*
Adrenal glands	1.9 ± 0.2	0.8 ± 0.2*
Kidneys	1.9 ± 0.0	0.7 ± 0.1***
Spleen	1.6 ± 0.0	2.1 ± 0.6
WAT	1.2 ± 0.3	0.8 ± 0.2
BAT	0.9 ± 0.1	0.5 ± 0.1*
Femur	0.5 ± 0.0	0.4 ± 0.0
Testicles	0.4 ± 0.0	0.3 ± 0.0
Pancreas	0.3 ± 0.1	0.3 ± 0.1
Skin + Hair	0.4 ± 0.0	0.7 ± 0.1**
Muscle	0.1 ± 0.0	0.1 ± 0.0
Tumor	0.4 ± 0.0	3.1 ± 0.3***

The SUV were determined from four PC3-P tumor-bearing mice in each group ± SEM. Significant differences with t-test: 48 h relative to 1 h values; * $p < 0.05$; ** $p < 0.01$; *** $p < 0.001$.

Table S2. Biodistribution of [^{177}Lu]Lu-DTPA-7F5 in PC3-P tumor-bearing mice, 1 and 48 h after injection, accumulation determined as ID/g tissue.

	1 h	48 h
Blood	30.3 ± 1.8	7.0 ± 0.3***
Heart	11.6 ± 1.1	3.0 ± 0.3***
Brain	0.9 ± 0.1	0.2 ± 0.0**
Lungs	15.2 ± 3.7	3.7 ± 0.2*
Liver	14.4 ± 1.8	20.6 ± 2.8
Adrenal glands	8.9 ± 1.5	3.7 ± 1.1*
Kidneys	8.5 ± 0.4	3.0 ± 0.4***
Spleen	7.6 ± 0.4	9.5 ± 2.9
WAT	5.4 ± 1.5	3.7 ± 1.1
BAT	4.1 ± 0.7	2.1 ± 0.2*
Femur	2.1 ± 0.2	1.7 ± 0.1
Testicles	1.7 ± 0.2	1.3 ± 0.0
Pancreas	1.6 ± 0.5	1.5 ± 0.5
Skin + Hair	1.6 ± 0.1	2.9 ± 0.2**
Muscle	0.6 ± 0.1	0.5 ± 0.0
Tumor	1.7 ± 0.1	13.6 ± 1.3***
Thyroid	4.1 ± 0.8	1.0 ± 0.3*
Stomach[a]	0.5 ± 0.1	0.6 ± 0.1
Intestine[a]	3.7 ± 0.5	4.3 ± 0.8
Urine[a]	11.1 ± 0.7	24.6 ± 0.8**

% ID/g tissue were determined from four PC3-P tumor-bearing mice in each group ± SEM.
[a]Data are expressed in %ID ± SEM.
Significant differences with t-test: 48 h relative to 1 h values; * $p < 0.05$; ** $p < 0.01$; *** $p < 0.001$.
